# 78 Transient neonatal Behçet’s disease: a case report

**DOI:** 10.1093/rheumatology/keac496.074

**Published:** 2022-10-07

**Authors:** Rouag Hatem, El-Ouaer Marwa, Dhouib Salma, Ben Mohamed Zaineb, Bedoui Ghofrane, Abdelmoula Siwar, Ben Hmida Hayet, Monastiri Kamel

**Affiliations:** Neonatal Intensive Care and Neonatal Medicine Department, Maternity and Neonatology Center, Fattouma Bourguiba University Hospital, Monastir, Tunisia

## Abstract

**Background:**

Behçet’s disease (BD) is a systemic vasculitis of unknown aetiology. The disease usually affects patients in the 3rd life decade and is rare in pediatrics. A very rare clinical form of Behçet's disease occurs during neonatal period.

**Observation:**

We report a case of a term neonate presenting with oral and genital ulcerations appeared at the age of 7 days. The mother had a history of Behçet’s disease and antiphospholipid antibody syndrome (APLS) diagnosed at the age of 20 years, characterized by severe recurrent orogenital ulceration and complicated by thrombophlebitis and mycocarditis. She was treated with colchicine, corticoids during the attacks and an antiplatelet treatment. Also, the siblings of the neonate (2 sisters and one brother) had the same symptoms in the neonatal period and developed disfiguring scars since they were treated as herpes by antiviral therapy. Family history, clinical course and negative laboratory results, suggest the diagnosis of transient neonatal BD. The treatment with systemic and local corticoids was effective. The neonate didn’t develop scars, neurological or vascular symptoms. The antibodies anti-nuclear and HLAB51 were negative.

**Conclusion:**

Transient neonatal Behçet’s disease (BD) is a rare secondary neonatal autoimmune condition. The transmission from mother to fetus is still unclear. It is thought to be transmitted by an immune mechanism. However, the causative antibodies for neonatal BD have not been identified. Corticosteroid is recommended after diagnosis in order to avoid scars

